# Initial Decomposition Mechanism of 3-Nitro-1,2,4-triazol-5-one (NTO) under Shock Loading: ReaxFF Parameterization and Molecular Dynamic Study

**DOI:** 10.3390/molecules26164808

**Published:** 2021-08-09

**Authors:** Lixiaosong Du, Shaohua Jin, Pengsong Nie, Chongchong She, Junfeng Wang

**Affiliations:** 1 School of Materials Science & Engineering, Beijing Institute of Technology, Zhongguancun South Street 5, Beijing 100081, China; 3120185542@bit.edu.cn (L.D.); 3120195573@bit.edu.cn (P.N.); 3120195576@bit.edu.cn (C.S.); 2 State Key Laboratory of Explosion Science, Beijing Institute of Technology, Zhongguancun South Street 5, Beijing 100081, China

**Keywords:** 3-Nitro-1,2,4-triazol-5-one (NTO), ReaxFF-lg parameterization, reactive molecular dynamic, shock Hugoniot state, shock-induced initial decomposition mechanisms

## Abstract

We report a reactive molecular dynamic (ReaxFF-MD) study using the newly parameterized ReaxFF-lg reactive force field to explore the initial decomposition mechanism of 3-Nitro-1,2,4-triazol-5-one (NTO) under shock loading (shock velocity >6 km/s). The new ReaxFF-lg parameters were trained from massive quantum mechanics data and experimental values, especially including the bond dissociation curves, valence angle bending curves, dihedral angle torsion curves, and unimolecular decomposition paths of 3-Nitro-1,2,4-triazol-5-one (NTO), 1,3,5-Trinitro-1,3,5-triazine (RDX), and 1,1-Diamino-2,2-dinitroethylene (FOX-7). The simulation results were obtained by analyzing the ReaxFF dynamic trajectories, which predicted the most frequent chain reactions that occurred before NTO decomposition was the unimolecular NTO merged into clusters ((C_2_H_2_O_3_N_4_)_n_). Then, the NTO dissociated from (C_2_H_2_O_3_N_4_)_n_ and started to decompose. In addition, the paths of NO_2_ elimination and skeleton heterocycle cleavage were considered as the dominant initial decomposition mechanisms of NTO. A small amount of NTO dissociation was triggered by the intermolecular hydrogen transfer, instead of the intramolecular one. For α-NTO, the calculated equation of state was in excellent agreement with the experimental data. Moreover, the discontinuity slope of the shock-particle velocity equation was presented at a shock velocity of 4 km/s. However, the slope of the shock-particle velocity equation for β-NTO showed no discontinuity in the shock wave velocity range of 3–11 km/s. These studies showed that MD by using a suitable ReaxFF-lg parameter set, could provided detailed atomistic information to explain the shock-induced complex reaction mechanisms of energetic materials. With the ReaxFF-MD coupling MSST method and a cheap computational cost, one could also obtain the deformation behaviors and equation of states for energetic materials under conditions of extreme pressure.

## 1. Introduction

The decomposition mechanism of 3-Nitro-1,2,4-triazol-5-one (NTO) [1] under different conditions obviously influences the safety performance of NTO-based insensitive ammunition (IM) [2,3,4,5]. The thermal-induced decomposition kinetic process and burning characteristics of NTO [6,7,8,9,10,11] have been studied via various experimental techniques. In addition, the theoretical works concerning the electron structures and possible decomposition paths of NTO [12,13,14] were also reported. However, the atom-scale understanding about the shock-induced chemistry reaction of NTO still remain unclear.

Due to the progress of simulation technology, non-equilibrium ReaxFF reactive molecular dynamics [15,16,17] provided the high possibility to investigate the shock-induced decomposition reaction of NTO at the molecular level. ReaxFF [15], developed by van Duin, is a bond-order-dependent reactive force field based on massive quantum mechanics (QM) calculation, which enables the accurate simulation of energetic materials under a realistic condition with a cheap computation. The shock-induced decomposition mechanisms of typical energetic materials (such as RDX [17], HMX [18], TATB [18], PETN [19], and TNT [20]) revealed by the ReaxFF molecular dynamics were in good agreement with the data from the experimental observations. To date, unfortunately, the poor transferability of the existing ReaxFF force fields [17,18,21,22] has led to no suitable ReaxFF potentials for simulating the thermal- and shock-induced chemistry reaction processes of NTO, hexanitrohexaazaisowurtzitane (CL-20), and dihydroxylammonium 5, 5′-bistetrazole-1, 1′-diolate (TKX-50). Therefore, developing a new ReaxFF parameter set to describe the physical and chemical properties of NTO, CL-20, and TKX-50 is a basic work for the exploration of the atom-scale decomposition chemistry of those energetic molecules.

In this study, we performed extensive QM calculations to parameterize a new ReaxFF-lg force field for 3-Nitro-1,2,4-triazol-5-one (NTO). Then, we used the new ReaxFF-lg parameters to model the shock-induced decomposition process of NTO crystal. The shock Hugoniot states and kinetic decomposition mechanism in the shock velocity range of 6–11 km/s were studied to obtain a complete map of the shock-induced initial decomposition mechanisms of NTO under extreme conditions.

## 2. Method

### 2.1. ReaxFF Parameterization

The general ReaxFF potential functions partition the total energy into the contributions as:

(1) $$\begin{array}{ll} E_{Reax}=E_{bond} & +E_{lp}+E_{over}+E_{under}+E_{val}+E_{pen}+E_{coa}+E_{c2}\\ & +E_{triple}+E_{tors}+E_{conj}+E_{H-bond}+E_{vdW}\\ & +E_{coulomb} \end{array}$$

The low-gradient model (*E_lg_*) [21] is the long-range dispersion-correction for *E_Reax_* to improve the descriptions of molecule crystalline:

(2) $$E_{Reaxlg}=E_{Reax}+E_{lg}$$

The critical difference between ReaxFF and unreactive force fields is that the bond order (*BO*′*_ij_*) decides the connectivity for two atoms, instead of a fixed bond. The *BO*′*_ij_* (Equation (3)) is updated in every iteration and allows for the breaking and recreation of chemical bonds during the molecular dynamics. Therefore, all covalent interactions, such as *E_bond_* and *E_val_*, are expressed in the terms of the corrected *BO*′*_ij_*.

(3) $$\begin{array}{c} BO_{ij}^{\prime }=BO_{ij}^{\sigma }+BO_{ij}^{\pi }+BO_{ij}^{\pi \pi }\\ =e^{\left[P_{bo1}{\left(\frac{r_{ij}}{r_{0}^{\sigma }}\right)}^{p_{bo2}}\right]}+e^{\left[P_{bo3}{\left(\frac{r_{ij}}{r_{0}^{\pi }}\right)}^{p_{bo4}}\right]}+e^{\left[P_{bo5}{\left(\frac{r_{ij}}{r_{0}^{\pi \pi }}\right)}^{p_{bo6}}\right]} \end{array}$$

To consider the Coulomb interactions between all atom pairs in the system, the atomic charges are updated using the Electron Equilibration Method (EEM) [23] approach at each iteration step. A similar method, called the QEq [24] charge derivation approach, is also suitable for calculating the atomic charge distribution during the dynamics. The atomic charge calculation in ReaxFF is the most time-consuming step, so the calculation cost of ReaxFF-MD is about two orders of magnitude higher than that of the classic non-reaction force field. However, the computation costs of ReaxFF are still far less than the ab-initio molecular dynamics.

**Figure 1:**
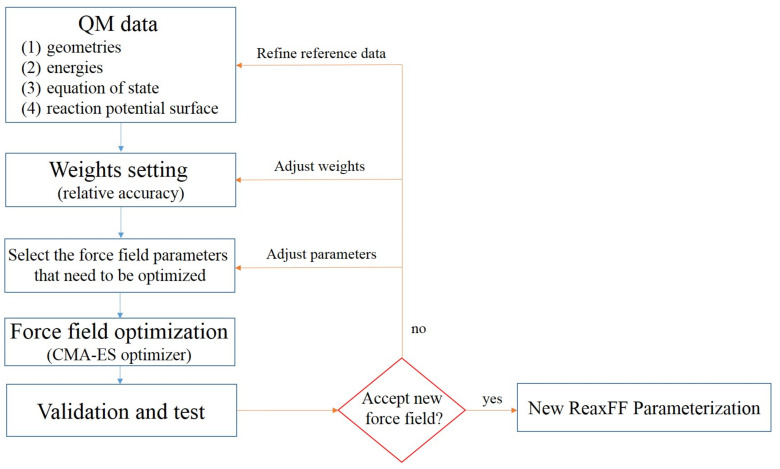
Workflow of the ReaxFF parameterization.

The parameters of the ReaxFF are usually fitted from massive QM data and experimental values. In this study, the parametrization workflow of a new force field consisted of the following steps (Figure 1):

(1) Generation of the reference QM data (training set). In this study, the DFT methods with Grimme dispersion correction [25] were used to obtain the bond dissociation curves, valence angle bending curves, dihedral angle torsion curves, and unimolecular decomposition paths [13,26,27] of NTO, RDX, and FOX-7, respectively. Other important properties, such as the geometries (bond length, valence angles, and dihedral angles), interaction energy of dimers by hydrogen bonds and vdW interaction, dissociation potential energy surfaces of small molecules, cohesive energy, and crystal equations of state were also added to the training set. The B3LYP-D3BJ and M062X-D3 functional [28] were used to optimize the minimum points and transition state structures and predict the reaction energies. Moreover, the dimers’ interaction energy and crystal equation of state were evaluated by the PWPB95-D3BJ approach [29] and PBEsol [30] with TS dispersion correction [31], respectively. Finally, the training set contained over 40 chemical reactions and over 1700 molecules for characterizing the atomic interactions under various environments. For detailed information about the training data, please see the Supplementary Materials.

(2) The weights (*σ*) assignment of the objective function between the ReaxFF prediction and QM data. The objective function has the form:

(4) $$Error=\overset{n}{\underset{i=1}{\sum }}{\left[\frac{x_{i,TS}-x_{i,ReaxFF}}{\sigma_{i}}\right]}^{2}$$

where the sum runs over all the training set entries. Each difference between the reference property (*x_i,TS_*) and the ReaxFF value (*x_i,ReaxFF_*) is weighted individually via the *σ_i_*. In this study, the properties of NTO were assigned smaller weights to highlight the importance during the force field optimization.

(3) The Covariance Matrix Adaption Evolution Strategy (CMA-ES) optimizer of AMS2020 [32], coupled with the RiPSOGM global optimization algorithm [33], was used to fit the new ReaxFF parameter set. At the beginning of the optimization, the ReaxFF parameters from reference [21] were chosen as a good initial force field.

(4) The crystal properties and reaction energy profiles were considered as the most important items for validating the new force field. Other properties, such as the enthalpies of sublimation and geometries, were also used for the evaluations.

### 2.2. Molecular Dynamic Simulations

The shock-induced decompositions of β-NTO at various shock wave velocities (6–11 km/s) were simulated by the multi-scale shock technique (MSST) [34], coupled with ReaxFF-MD. The single crystal of β-NTO, obtained from the Cambridge Crystallographic Data Centre (CCDC No. 166510), was expanded to a 6 × 6 × 6 supercell that used as the initial state of the 3D periodic box. In the simulation, a 1.5 ps isothermal isochoric (NVT ensemble, langevin thermostat, 50 fs damping parameter) MD was firstly conducted to relax the atomic positions of the initial state. Then, a shock wave along the lattice vector direction *a* was loaded to study the non-equilibrium evolution of the modelling system. For each simulation, the dynamic evolution time was 50 ps, and the integral step was set to 0.1 fs. The 0.3 bond’s cutoff was used for the analysis of intermediates and products. All dynamic simulations were performed on the LAMMPS [35] package. For post-processing of the dynamic trajectory, the python scripts embedded in the MAPS [36] package were used.

## 3. Result and Discussion

### 3.1. Parameterization and Verification

#### 3.1.1. New Reaxff-Lg Parameterization for NTO

Based on the massive reference QM data and experimental values, we obtained multiple ReaxFF-lg parameter sets of NTO from training. Then, one force field with the best performance was chosen as the new ReaxFF-lg potentials of NTO. This ReaxFF-lg parameters are presented in the Supplemental Materials.

#### 3.1.2. Description of the Crystal Properties

To validate the chosen force field, we studied the equilibrium structure (3 × 3 × 3 supercell) of β-NTO by a 1 ps NVT-MD and subsequent 5 ps NPT-MD under standard conditions (298 k and 1 atm). The damping parameters were 50 fs for the thermostat and 500 fs for the barostat. After the 6 ps simulation, the calculated lattice constants were compared to the experimental data. An additional 25 ps NVT-MD under a lower temperature (5 k) was performed to calculate the heat of sublimation (ΔH_sub_). In order to measure the differences between the equilibrium structures and initial geometries during this NVT-MD, we also calculated the average values of the root-mean-square deviations (RMSD) for the atomic position by considering all the dynamic trajectories. For comparison, the calculation results by MD using the original and new ReaxFF-lg parameters, are listed in Table 1.

As shown in Table 1, the lattice constants and density were predicted by the original and new ReaxFF-lg force fields. Obviously, the results of the new ReaxFF-lg were closer to the experimental density. In addition, the error of ΔH_sub_, predicted by the original ReaxFF-lg and obtained by the experiment, was more than 40% while it was reduced to a very low level using the new ReaxFF-lg. Moreover, the RMSD of β-NTO and single-molecule NTO, calculated by the new ReaxFF-lg, showed lower values than that of the original ReaxFF-lg. This fact indicated that the inner molecule vibration of the crystal predicted by the new ReaxFF-lg MD was more similar to the original unit cell structure. Another noticeable phenomenon observed from the original ReaxFF-lg MD was that the hydrogen atoms in the nitrogen heterocycle ring were not on the same plane as the ring, exhibiting a bad reproduction of original single crystal structure. Fortunately, this phenomenon did not happen in the new ReaxFF-lg MD. For detailed information about this phenomenon, a few animations are provided in the Supplemental Materials for visualizing the differences.

**Figure 2:**
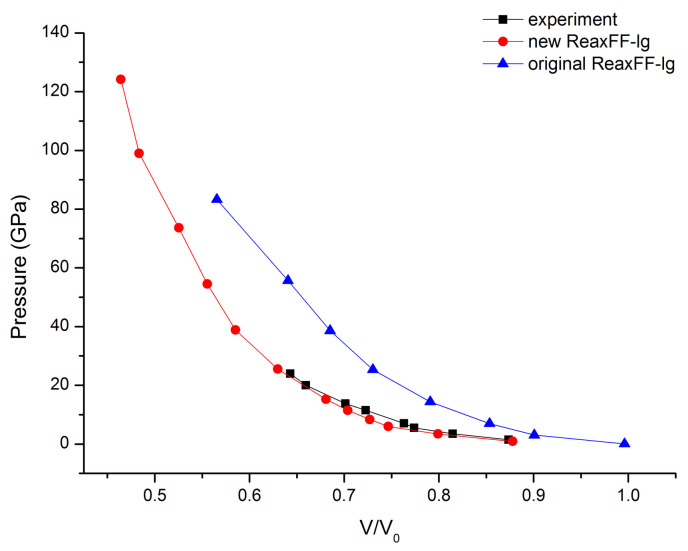
Comparisons of the compression equation of state for α-NTO.

For investigating the prediction ability of the new ReaxFF-lg force field on the compression properties of the NTO crystals, the equation of states of α-NTO were calculated by ReaxFF-MD, coupled with the MSST methods (mentioned in Section 2.2). The calculated data are drawn in Figure 2, accompanied by the experimental data [38]. More shock Hugoniot data are provided in the Supplemental Materials.

From Figure 2, the calculated results from the new force field are in good agreement with the experimental values, which means that the new ReaxFF-lg parameters accurately reproduced the shock compression properties of the α-NTO crystals. While the equation of state (EOS) data of α-NTO were not included in the training data, the new ReaxFF-lg parameters still successfully predicted the equation of states for α-NTO between 0–25GPa with an excellent accuracy, indicating the good transferability of the new force field. Therefore, the new ReaxFF-lg parameters were suitable for simulating the deformation behavior of the NTO crystals during shock loading. However, the results from the original ReaxFF-lg MD overestimated the pressure of the Hugonito state, indicating that the original ReaxFF-lg almost failed to model the compression EOS of the NTO crystals.

#### 3.1.3. Description of the Potential Energy Surface

**Figure 3:**
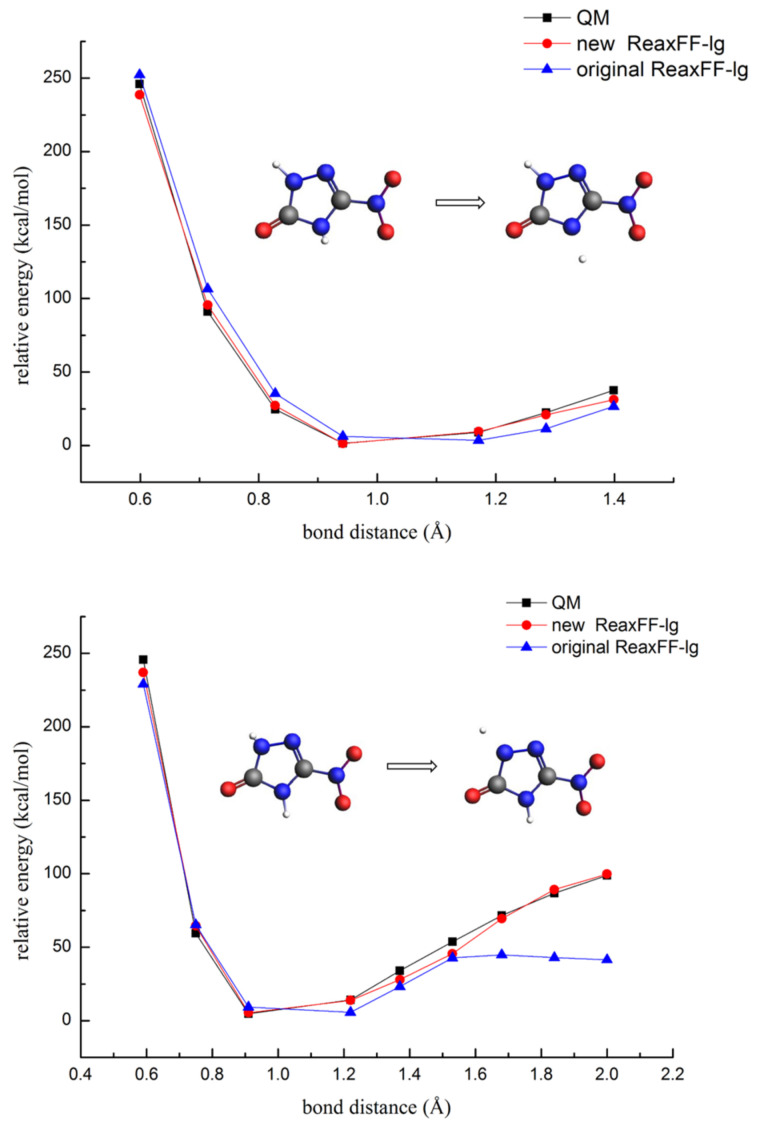
The dissociation energies for the two N-H single bonds at the skeleton ring of NTO (gray—carbon atom; white—hydrogen atom; red—oxygen atom; blue—nitrogen atom).

**Figure 4:**
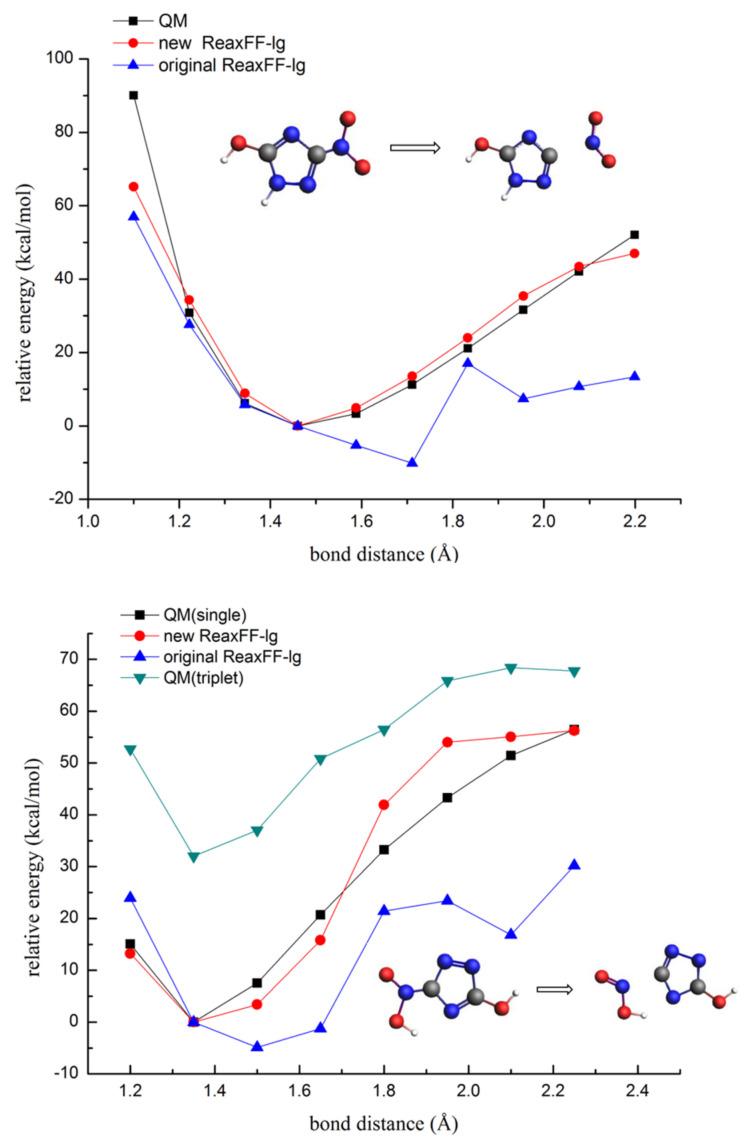
The dissociation energies of the C-N single bond during NO_2_ and HONO elimination (gray—carbon atom; white—hydrogen atom; red—oxygen atom; blue—nitrogen atom).

The hydrogen transfer reaction and nitro group dissociation were vital for triggering the decomposition of the NTO molecule. In this paper, the bond dissociation energy profile of N-H and C-NO_2_ single bond prediction by the QM and ReaxFF methods were considered for verification of the new ReaxFF-lg (shown in Figure 3 and Figure 4).

From Figure 3, the prediction energy curves for the dissociation of the hydrogen atom attached to the ring by the new ReaxFF-lg obtained a good consistency with the QM data, indicating that the new ReaxFF-lg force fields could well reproduce the energies of the proton dissociation process. While the original ReaxFF-lg could predict the dissociation energy of one hydrogen atom, it could not describe the energy increases for another hydrogen atom dissociation when the N-H bond length exceeded 1.5 Å.

From Figure 4, the original ReaxFF-lg failed to describe the NO_2_ and HONO dissociation reaction energy profiles, thus poorly describing the initiation decomposition step of the NTO molecule. On the contrary, the predicted energy curves of the C-N bond broken by the new ReaxFF-lg were more approximate to the QM curves, indicating that the new ReaxFF-lg significantly improved the prediction accuracy of the energy changes in the NO_2_ and HONO dissociation. It was also noticeable that the prediction energy at the C-N bond distances of 1.8–2.1 Å for the HONO dissociation by the new ReaxFF-lg were between the energy curves of the single and triplet states. Thus, we thought the fitting errors were acceptable for the HONO dissociation, and the fitting curves also gave a qualitatively correct energy profile.

**Figure 5:**
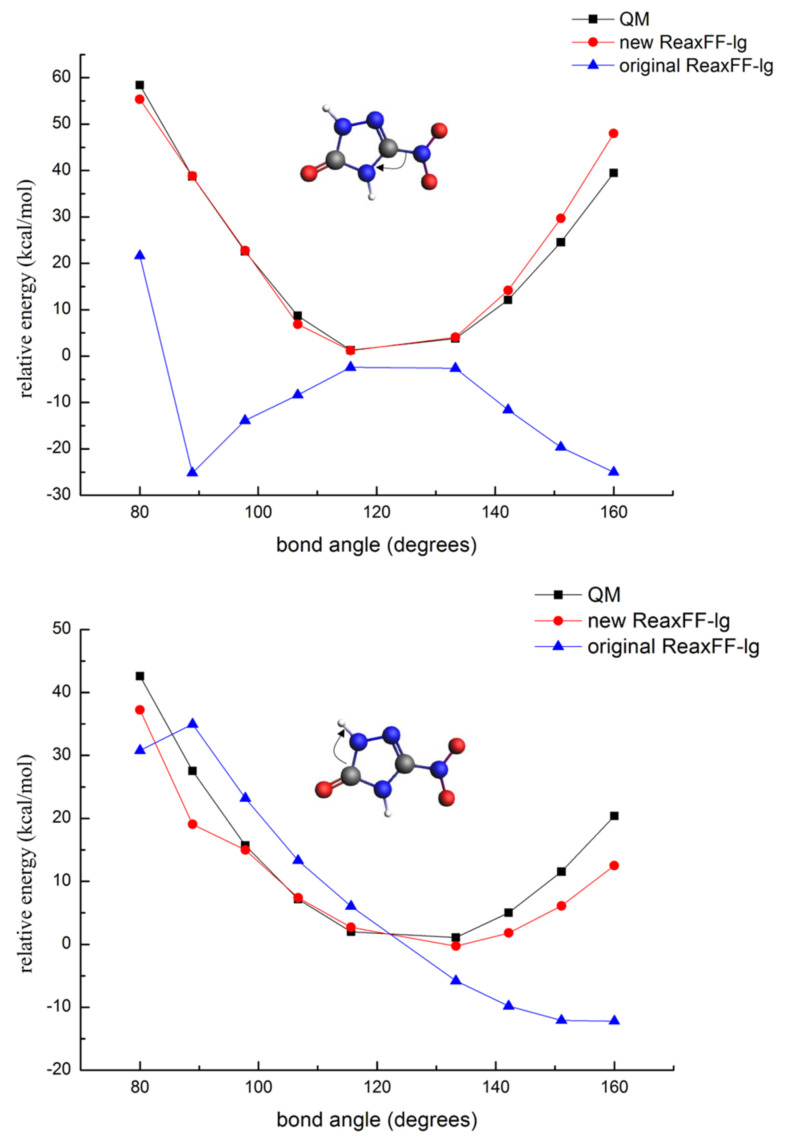
Energy barriers for the distortion of the N-C-N and C-N-H bond angles in NTO (gray—carbon atom; white—hydrogen atom; red—oxygen atom; blue—nitrogen atom).

**Figure 6:**
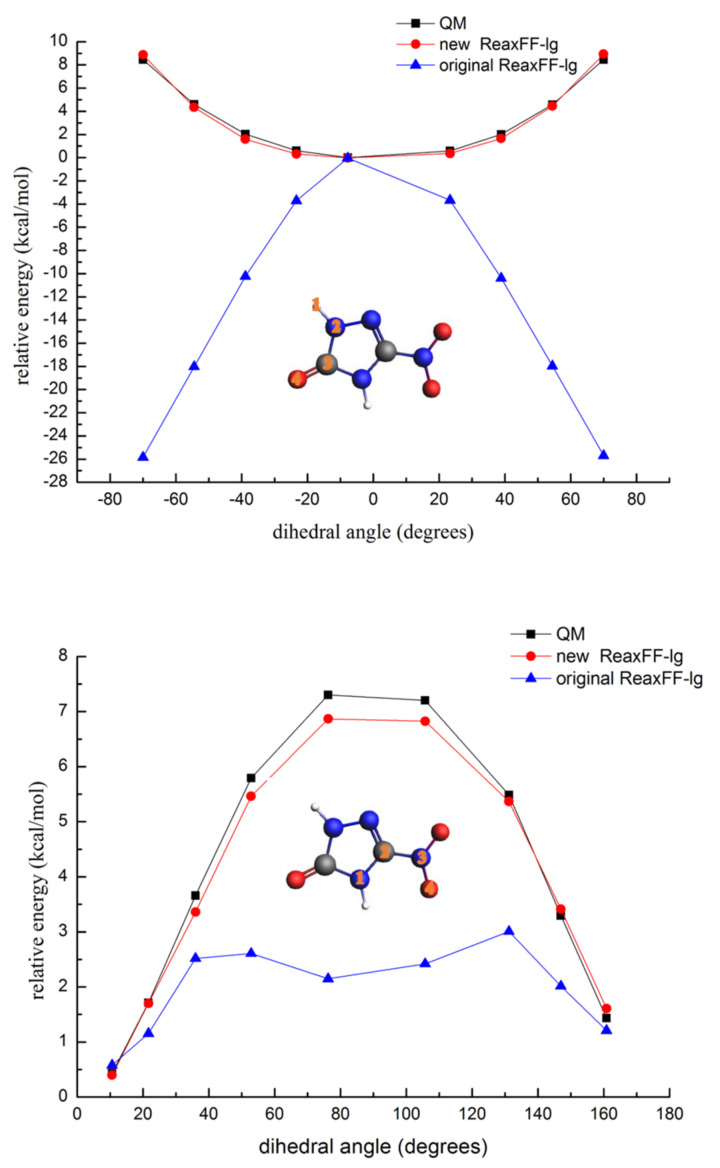
Rotational energy barriers for the H-C-N-O and N-C-N-O dihedral angles in NTO (gray—carbon atom; white—hydrogen atom; red—oxygen atom; blue—nitrogen atom).

To verify whether the ReaxFF-lg force field can accurately predict the valence angle bending and dihedral angle rotation energy barriers in the NTO molecule, the potential energy surfaces of the N-C-N, C-N-H, H-C-N-O, and N-C-N-O angles were calculated using the ReaxFF-lg and QM methods. These energy curves are shown in Figure 5 and Figure 6.

As shown in Figure 5 and Figure 6, the barriers of the N-C-N bond angle distortion and dihedral angles rotation calculated by the original ReaxFF-lg showed unacceptable errors, indicating that the original force field was completely unable to describe the angle potential energy surfaces of NTO. However, the energy curves obtained from the new ReaxFF-lg showed a good reproduction of the QM data. Thus, the new ReaxFF-lg could accurately predict the energy barriers of the valence angle distortion and dihedral angle rotation for the NTO molecule.

### 3.2. Shock-Induced Chemistry of NTO

#### 3.2.1. Shock Hugoniot State

For unreacted energetic materials, the shock wave velocity (*U_s_*) and particle velocity (*U_p_*) usually have the following linear relationship:

(5) $$U_{s}=C_{0}+SU_{p}$$

where *C*_0_ and *S* are constants. Additionally, the pressures (*P*) of the shock compression are calculated by:

(6) $$P=\rho_{0}U_{s}U_{p}$$

where ρ_0_ is the initial density.

**Figure 7:**
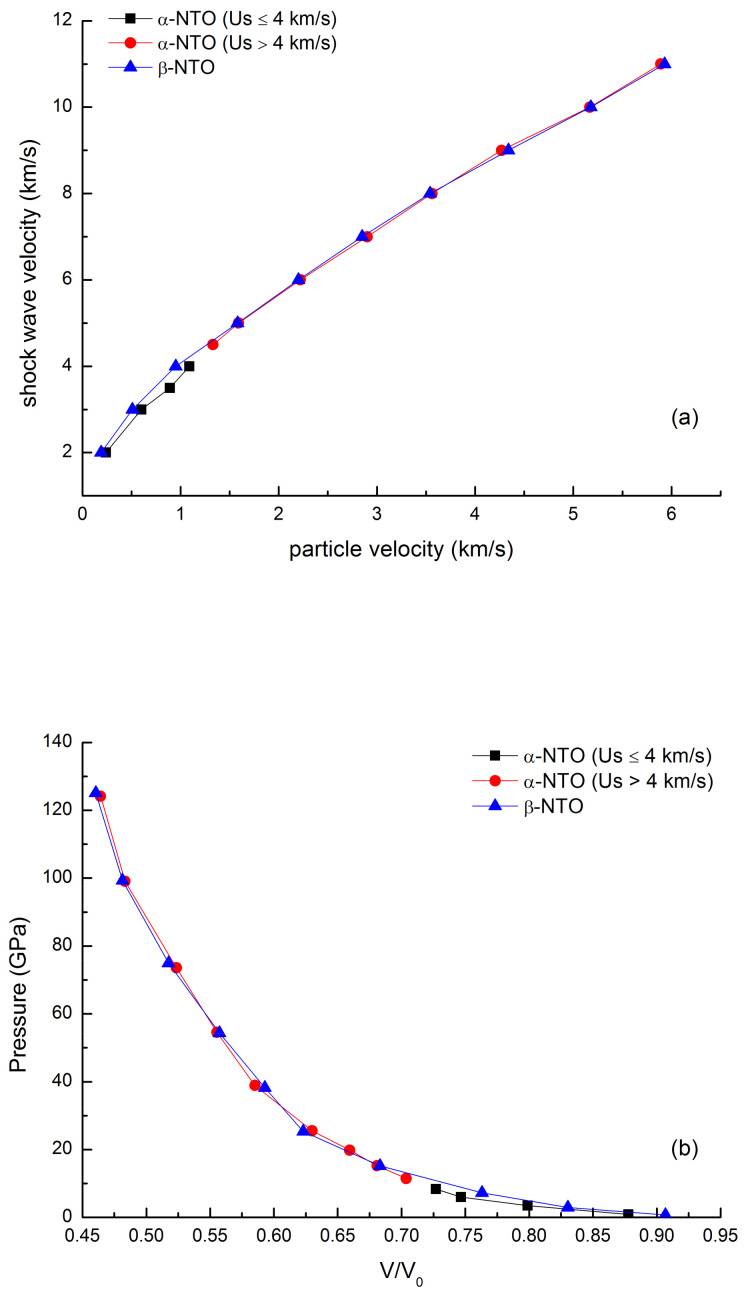
Shock Huoniot state data of α-NTO and β-NTO. (**a**) shock velocity and particle velocity relation; (**b**) pressure via relative volume along Hugoniot line.

Since the calculated equation of state for α-NTO in Section 2.2 was a verification of the new force field’s good description ability of the shock compression properties of NTO crystals, we expanded the shock hugoniot data of α-NTO and calculated the *U_s_*–*U_p_* data of β-NTO. These values are listed in Table 2, and the pressure-volume curves are shown in Figure 7.

From Figure 7a, when the shock wave velocity is 4 km/s, there is an obvious inflection point at the curves of α-NTO, showing that the slope of equation 5 for α-NTO had different values after the inflection point. Engelke [39] reported that the energetic molecules containing aromatic rings showed discontinuities in the principal-shock Hugoniot. Our simulation results for α-NTO also showed the same characteristics. Thus, the data before and after the inflection point of α-NTO were fitted by the least squares method to obtain the corresponding C_0_ and S. However, for β-NTO, the slope of equation 5 was no discontinuity in the range of the shock wave velocity of 3–11 km/s, indicating that the particle velocity and the shock velocity maintain a good linear relationship.

(7) $$\alpha \text{-}NTO:\;\;\;U_{s}=1.5032+2.3005U_{p\;}(2\;\mathrm{km}/s\leq U_{s}\leq 4\mathrm{km}/s,\;R^{2}=0.9912)$$

(8) $$\alpha \text{-}NTO:\;\;U_{s}=2.8039+1.4136U_{p\;}(4\;\mathrm{km}/s<U_{s}\leq 11\;\mathrm{km}/s,\;R^{2}=0.9966)$$

(9) $$\beta \text{-}NTO:\;\;U_{s}=2.6449+1.4469U_{p\;}(3\;\mathrm{km}/s\leq U_{s}\leq 11\;km/s,\;R^{2}=0.9940)$$

From Figure 7b, it was clear that the pressure-relative volume curves of those NTO crystals showed little differences, indicating that NTO crystals in α and β phase showed similar compression properties. This phenomenon may be due to the similar molecular arrangement characteristics and smaller density differences between the two crystals.

#### 3.2.2. Initial Decomposition Mechanism

To understand the ideal detonation behavior of NTO, we mainly consider the evolution and reaction mechanism of NTO under strong shock waves (shock velocity >6 km/s). The massive reactions occurred during the ReaxFF-MD; therefore, we pointed out the cluster formation and initial decomposition paths of NTO, while neglecting the final product formation mechanism.

**Figure 8:**
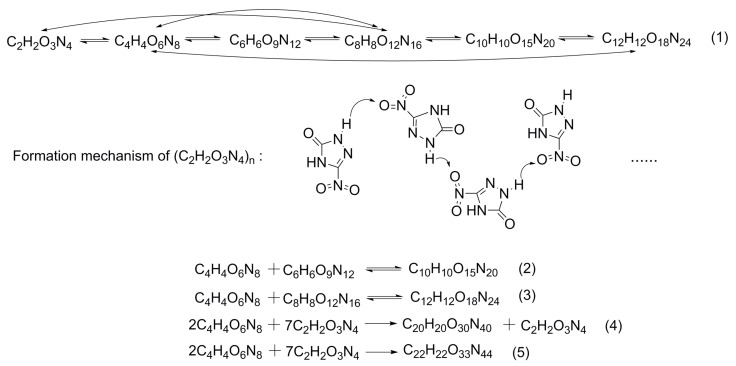
(C_2_H_2_O_3_N_4_)_n_ formation.

(1) Cluster formation: the aggregation of the NTO molecules was caused by the strong compression during the shock wave front propagating across the NTO crystal. This was manifested as the NTO molecules merging into dimers, trimers, and even larger (C_2_H_2_O_3_N_4_)_n_. Figure 8 illustrates the formation process of the NTO clusters.

The chemical reaction (1), listed in Figure 8, was the common chain reaction obtained from all simulations, of which the NTO dimer formation was the most frequently and earliest occurring reaction. From ReaxFF-MD, the mechanism of (C_2_H_2_O_3_N_4_)_n_ formation was that the intermolecular NH…O hydrogen bonds were broken by shock compression, and then the oxygen atom of the nitro group attacked the hydrogen atom of the heterocyclic ring to produce the O-H single bond. Thus, the hydrogen atom was the bridge to form NTO clusters in the early evolution. In addition, (C_2_H_2_O_3_N_4_)_n_ underwent a direct polymerization reaction (Reactions (4) and (5) in Figure 8) to form larger clusters when the shock wave velocity was higher than the Chapman-Jouguet detonation velocity of NTO (~8 km/s). Furthermore, the higher the shock wave velocity, the higher the degree of NTO polymerization.

**Figure 9:**
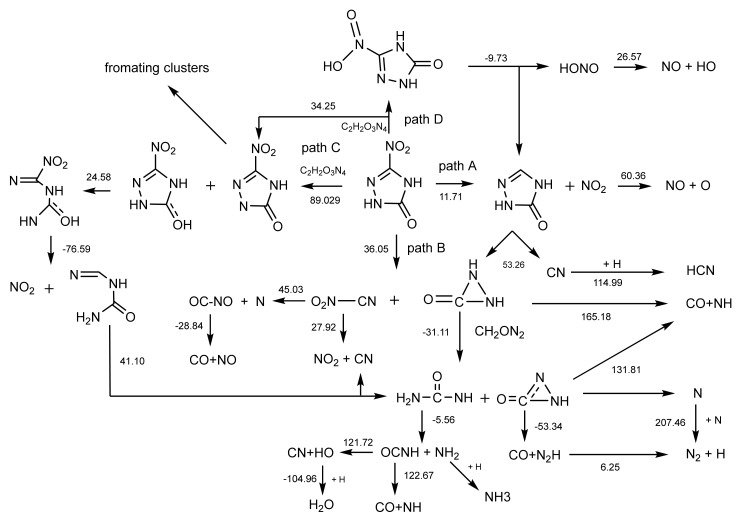
The initial decomposition paths of NTO.

(2) The initial decomposition paths of NTO: an interesting phenomenon was that almost all reactants were forming into (C_2_H_2_O_3_N_4_)_n_ clusters. After that, the unimolecular NTO was dissociated from the (C_2_H_2_O_3_N_4_)_n_ clusters and started to decompose along the paths shown in Figure 9. In addition, the Gibbs free energy changes of the reaction at high temperature and pressures (calculated by M062X-D3/def2-tzvp) are marked in Figure 9.

From Figure 9, there were four decomposition paths for unimolecular NTO. The NO_2_ elimination (path A) was found to be the early and frequently decomposition path, followed by heterocycle cleavage, thus forming the unstable intermediate (CH_2_ON_2_) and radicals CN. In addition, heterocycle cleavage (path B) and hydrogen transfer (paths C and D) both took place later and less frequently than path A because of their relative higher Gibbs free energy barriers. However, the occurring frequency of NTO decomposition along path B was far more than the intermolecular hydrogen transfer reactions.

Path A showed that NTO was first broken into the NO_2_ and C_2_H_2_ON_3_ ring, where the ring was split into CN and a three-atom nitrogen heterocycle (CH_2_ON_2_). Then, the C_2_H_2_ON_2_ with a high activity could spontaneously undergo an intermolecular proton transfer to form CH_3_ON_2_ and CHON_2_, and CHON_2_ converted into CO and N_2_. The CH_2_ON_2_ was also the product of the first decomposition step along path B and was accompanied by the O_2_N-CN formation. The intermediate O_2_N-CN could not only form NO_2_ and CN by breaking the C-N bond, but also dissociate a nitrogen atom and rearrange to form O=CNO. The latter one spontaneously decomposed into CO and NO. The decomposition along path C was triggered by the hydrogen transfer towards carbonyl. The stable product C_2_H_2_O_3_N_4_ of path C broke the N-N bond of the heterocycle, then NO_2_ elimination happened immediately, thus forming the C_2_H_3_ON_3_. The decomposition paths A, B, and C all produced the same molecule, CH_3_ON_2_, and the C-NH_2_ bond of this molecule easily lost its connection to produce OCNH and NH_2_. The reaction along path D was another different hydrogen transfer situation. It should have been the case that the –NO_2_ group captured surrounding protons due to its strong electronegativity. However, the unstable intermediate, C_2_H_2_ON_3_-HONO, automatically dissociated into HONO and C_2_H_2_ON_3_, undergoing decomposition along path A at last.

Wang et al. explored the unimolecular decomposition mechanism of NTO by first-principle calculation [13]. They proposed that the hydrogen transfer at the nitrogen heterocycle and NO_2_ dissociation were the initial decomposition steps of single NTO. However, our study did not find the intramolecular hydrogen transfer during MD; instead, we found the intermolecular hydrogen transfer paths (path C and D in Figure 9). Moreover, we found a new decomposition path: skeleton heterocycle cleavage (path B in Figure 9) under shock. On the other hand, the calculation results of Wang et al. [13] indicated that the energy barriers of hydrogen transfer were about 20 kcal/mol lower than that of NO_2_ elimination. The opposite results that we acquired showed that the high temperature and high stress inside the NTO crystal significantly reduced the NO_2_ elimination energy barrier and the NTO intramolecular stability. The spontaneous polymerization of NTO under a strong shock wave (shown in Figure 8) also inhibited the intramolecular hydrogen transfer reaction. Because of the contribution of the two parts, the NO_2_ elimination and heterocycle cleavage became the primary decomposition pathways of NTO under shock wave.

## 4. Conclusions

A new ReaxFF-lg force field was customized to describe the properties of 3-Nitro-1,2,4-triazol-5-one (NTO). This force field was trained from massive quantum mechanics data and experimental values, especially including the bond dissociation curves, valence angle bending curves, dihedral angle torsion curves, and unimolecular decomposition paths of NTO. Through verification, the new ReaxFF-lg parametrization was proved to be a suitable reactive force field for modelling the physical and chemistry properties of NTO.

Reactive molecular dynamic (ReaxFF-MD) simulations with the new ReaxFF-lg force field were performed to explore the initial decomposition mechanism of NTO under shock (shock velocity >6 km/s). These simulations showed that the most frequent chain reactions occurred before NTO decomposition was the single NTO molecules merged into dimers, trimers, and even large (C_2_H_2_O_3_N_4_)_n_ clusters.

Then, the NTO single molecule started to decompose after dissociating from the (C_2_H_2_O_3_N_4_)_n_ clusters. The paths of NO_2_ elimination and skeleton heterocycle cleavage were considered as the dominant initial decomposition mechanisms of NTO. A small amount of NTO decomposition was triggered by the intermolecular hydrogen transfer, instead of the intramolecular one. Moreover, the NTO crystals in α and β phase showed similar compression properties due to the similar molecular arrangement and smaller density differences between the two crystals.

These studies showed that MD, using a suitable ReaxFF-lg potential, provided detailed atomistic information to explain the shock-induced complex reaction mechanisms of energetic materials. By the ReaxFF-MD coupling MSST method, the deformation behaviors and equation of states of energetic materials under conditions of extreme pressure could be obtained with a cheaper computational cost.

## Figures and Tables

**Table 1:** The lattice constants, density, heat of sublimation, and root-mean-square deviations of β-NTO.

Item		New ReaxFF-lg	Original ReaxFF-lg [21]	Experiment
a		9.317	9.380	9.326
b		5.445	5.482	5.450
c		8.851	9.093	9.040
α		90.0	90.0	90.0
β		101.47	101.47	101.47
γ		90.0	90.0	90.0
density (g/cm^3^)		1.926	1.885	1.918
density error (%)		−0.417	−1.721	−
ΔH_sub_ (kcal/mol)		25.62	39.89	23.42–28.65 [37]
RMSD (Å)	crystal	0.204	0.660	−
	molecule	0.063	0.247	−

**Table 2:** The shock Hugoniot state of α-NTO and β-NTO.

U_s_ (km/s)	*U_p_* (km/s)		*V*/*V_0_*		*P* (GPa)	
	α-NTO	β-NTO	α-NTO	β-NTO	α-NTO	β-NTO
2	0.24	0.19	0.88	0.91	0.92	0.73
3	0.6	0.51	0.80	0.83	3.45	2.93
4	1.09	0.95	0.73	0.76	8.35	7.29
5	1.59	1.58	0.68	0.68	15.23	15.16
6	2.22	2.20	0.63	0.63	25.51	25.32
7	2.90	2.85	0.59	0.59	38.88	38.27
8	3.56	3.54	0.56	0.56	54.55	54.32
9	4.27	4.34	0.53	0.52	73.61	74.92
10	5.17	5.18	0.48	0.48	99.03	99.35
11	5.89	5.93	0.46	0.49	124.15	125.11

Note: *V*/*V*_0_ is the relative volume.

## Data Availability

The full training-data files can be requested by contacting the author. Two email address are provided here: 3120185542@bit.edu.cn and dulixiaosong@163.com.

## Supplementary Materials

The following are available online. The Supplementary Materials produced the detailed information about the methods used in this study. The full training-data files can be requested by contacting the author. Figure S1.: N-H bond distance during NVT-MD.; Figure S2.: O=C-N-H bond distance during NVT-MD.; Table S1.: Shock Hugoniot data of α-NTO.

## Sample Availability

Samples of the compounds used in this study are available from the authors.
