# Supplementary material for: Initial Decomposition Mechanism of 3-Nitro-1,2,4-triazol-5-one (NTO) under Shock Loading: ReaxFF Parameterization and Molecular Dynamic Study
Source: Molecules. 2021 Aug 9;26(16):4808. doi: 10.3390/molecules26164808 (PMC8400475; doi:10.3390/molecules26164808)
Supplement: Supplementary file 1 [file molecules-26-04808-s001.zip › molecules-1282262 SF.pdf]

Supplemental Materials

ReaxFF-lg Force Field parameters file

A standard format potential file named "NTO_ReaxFFlg" were provided for direct use in the lammps-REAXC and AMS-ReaxFF modules. The newly parameterized ReaxFF-lg reactive force filed was also as follows:

[lgDispersion=1] (NTO/RDX/FOX-7)

39 ! Number of general parameters

50.0000 !p_boc1 Eq(4c): Overcoordination parameter

9.4514 !p_boc2 Eq(4d): Overcoordination parameter

29.8953 !p_coa2 Eq(15): Valency angle conjugation

216.5421 !p_trip4 Eq(20): Triple bond stabilisation

12.2245 !p_trip3 Eq(20): Triple bond stabilisation

0.0000 !k_c2 Eq(19): C2-correction

1.0701 !p_ovun6 Eq(12): Undercoordination

7.5000 !p_trip2 Eq(20): Triple bond stabilisation

11.9083 !p_ovun7 Eq(12): Undercoordination

13.3822 !p_ovun8 Eq(12): Undercoordination

-10.9834 !p_trip1 Eq(20): Triple bond stabilization

0.0000 !Lower Taper-radius (must be 0)

10.0000 !R_cut Eq(21): Upper Taper-radius

2.8793 !p_fe1 Eq(6a): Fe dimer correction

33.8667 !p_val6 Eq(13c): Valency undercoordination

3.3976 !p_lp1 Eq(8): Lone pair param

1.0563 !p_val9 Eq(13f): Valency angle exponent

2.0384 !p_val10 Eq(13g): Valency angle parameter

6.1431 !p_fe2 Eq(6a): Fe dimer correction

6.9290 !p_pen2 Eq(14a): Double bond/angle param

0.0283 !p_pen3 Eq(14a): Double bond/angle param

0.0570 !p_pen4 Eq(14a): Double bond/angle param

-2.4837 !p_fe3 Eq(6a): Fe dimer correction

5.8374 !p_tor2 Eq(16b): Torsion/BO parameter

10.0000 !p_tor3 Eq(16c): Torsion overcoordination

1.8820 !p_tor4 Eq(16c): Torsion overcoordination

-1.2327 !p_elho Eq(26a): electron-hole interaction

2.1861 !p_cot2 Eq(17b): Conjugation if tors13=0

1.5591 !p_vdW1 Eq(23b): vdWaals shielding
0.0100 !Cutoff for bond order (*100)
4.8414 !p_coa4 Eq(15): Valency angle conjugation
3.5857 !p_ovun4 Eq(11b): Over/Undercoordination
38.6472 !p_ovun3 Eq(11b): Over/Undercoordination
2.1533 !p_val8 Eq(13d): Valency/lone pair param
0.5000 !X_soft Eq(25): ACKS2 softness for X_ij
1.0000 !d Eq(23d): Scale factor in lg-dispersion
5.0000 !p_val Eq(27): Gauss exponent for electrons
0.0000 !1 Eq(13e): disable undecoord in val angle
6.9784 !p_coa3 Eq(15): Valency angle conjugation
7 ! Nr of atoms; cov.r; valency;a.m;Rvdw;Evdw;gammaEEM;cov.r2;#
alfa;gammavdW;valency;Eunder;Eover;chiEEM;etaEEM;n.u.
cov r3;Elp;Heat inc.;bo131;bo132;bo133;softcut;n.u.
ov/un;val1;n.u.;val3,vval4
C 1.3675 4.0000 12.0000 1.9146 0.0719 0.7230 1.2318 4.0000
8.9734 82.3163 4.0000 31.0823 79.5548 5.1820 8.7292 0.0000
1.2104 0.0000 183.8108 2.9027 39.0332 9.3840 0.8563 0.0000
-1.4686 7.1588 1.0564 4.0000 2.9663 1.6357 0.1358 16.5114
3.5880 1.9440
H 0.6374 1.0000 1.0080 1.1505 0.0962 0.7696 -0.1000 1.0000
8.7946 10.6123 1.0000 0.0000 121.1250 3.7630 10.6939 1.0000
-0.1000 0.0000 58.4369 5.7596 1.6288 0.5012 1.0698 0.0000
-13.9534 3.2221 1.0338 1.0000 2.8793 1.2029 0.0760 10.2466
6.9880 1.2430
O 1.4324 2.0000 15.9990 1.9552 0.1646 0.8242 1.1567 6.0000
9.3281 36.0770 4.0000 29.5271 116.0768 8.7733 8.7821 2.0000
0.9909 14.7235 69.2921 11.1169 2.4235 0.2791 0.9745 0.0000
-1.8012 1.2515 1.0493 4.0000 2.9225 1.7173 0.1577 10.0123
516.8300 1.8440
N 1.1896 3.0000 14.0000 1.7009 0.1161 0.6899 1.1340 5.0000
9.7137 54.1099 4.0000 32.4758 100.0000 9.0046 5.5661 2.0000
1.0636 0.0276 127.9672 3.3235 1.4388 1.2228 0.9745 0.0000
-2.0509 1.0047 1.0183 4.0000 2.8793 2.0428 0.0248 15.8421

89.0970 1.8810
S 1.9647 2.0000 32.0600 2.0783 0.2176 1.0336 1.5386 6.0000
9.9676 5.0812 4.0000 35.1648 112.1416 6.5000 8.2545 2.0000
1.4703 9.4922 70.0338 8.5146 28.0801 8.5010 0.9745 0.0000
-10.0773 2.7466 1.0338 6.2998 2.8793 1.8000 0.0000 14.0000
180.0000 2.0783
Si 2.0276 4.0000 28.0600 2.2042 0.1322 0.8218 1.5758 4.0000
11.9413 2.0618 4.0000 11.8211 136.4845 1.8038 7.3852 0.0000
-1.0000 0.0000 126.5331 6.4918 8.5961 0.2368 0.8563 0.0000
-3.8112 3.1873 1.0338 4.0000 2.5791 0.0000 0.0000 0.0000
180.0000 2.2042
X -0.1000 2.0000 1.0080 2.0000 0.0000 1.0000 -0.1000 6.0000
10.0000 2.5000 4.0000 0.0000 0.0000 8.5000 1.5000 0.0000
-0.1000 0.0000 -2.3700 8.7410 13.3640 0.6690 0.9745 0.0000
-11.0000 2.7466 1.0338 4.0000 2.8793 0.0000 0.0000 0.0000
180.0000 2.0000
18 ! Nr of bonds; Edis1;LPpen;n.u.;pbe1;pbo5;13corr;pbo6
pbe2;pbo3;pbo4;n.u.;pbo1;pbo2;ovcorr
1 1 149.1452 104.9435 67.6027 0.3790 -0.3045 1.0000 30.4515 0.4283
2.0982 -0.3504 6.9035 1.0000 -0.1760 6.8796 1.0000 0.0000
1 2 163.6889 0.0000 0.0000 -0.4525 0.0000 1.0000 6.0000 0.5921
12.1053 1.0000 0.0000 1.0000 -0.0097 8.6351 0.0000 0.0000
2 2 169.8421 0.0000 0.0000 -0.3591 0.0000 1.0000 6.0000 0.7503
9.3119 1.0000 0.0000 1.0000 -0.0169 5.9406 0.0000 0.0000
1 3 127.8168 105.0412 85.4093 0.5488 -0.1742 1.0000 15.8765 0.5236
1.7981 -0.3079 7.0252 1.0000 -0.2875 4.9407 0.0000 0.0000
3 3 108.9631 158.3501 42.0558 0.1226 -0.1324 1.0000 28.5716 0.2545
1.0000 -0.2656 8.6489 1.0000 -0.1000 6.8482 1.0000 0.0000
1 4 99.6659 121.4879 63.0779 -0.7053 -0.0210 1.0000 34.8655 0.6011
0.0893 -0.4990 9.8542 1.0000 -0.1125 5.3153 1.0000 0.0000
3 4 86.2082 131.7776 77.1441 0.3765 -0.1062 1.0000 16.8836 0.1773
0.7026 -0.1906 8.4054 1.0000 -0.1632 7.4758 1.0000 0.0000
4 4 143.3590 73.1247 163.7844 0.4090 -0.1462 1.0000 14.0325 0.7971
0.6495 -0.1653 12.2959 1.0000 -0.0895 6.3891 1.0000 0.0000

```
2   3 198.7160   0.0000   0.0000  -0.4844   0.0000   1.0000   6.0000   0.3647
1.5475   1.0000   0.0000   1.0000  -0.1780   3.6037   0.0000   0.0000
2   4 302.9356   0.0000   0.0000  -0.7924   0.0000   1.0000   6.0000   0.2349
9.2278   1.0000   0.0000   1.0000  -0.1579   5.2076   0.0000   0.0000
1   5 128.7959  56.4134  39.0716   0.0688  -0.4463   1.0000  31.1766   0.4530
0.1955  -0.3587   6.2148   1.0000  -0.0770   6.6386   1.0000   0.0000
2   5 128.6090   0.0000   0.0000  -0.5555   0.0000   1.0000   6.0000   0.4721
10.8735   1.0000   0.0000   1.0000  -0.0242   9.1937   1.0000   0.0000
3   5   0.0000   0.0000   0.0000   0.5563  -0.4038   1.0000  49.5611   0.6000
0.4259  -0.4577  12.7569   1.0000  -0.1100   7.1145   1.0000   0.0000
4   5   0.0000   0.0000   0.0000   0.4438  -0.2034   1.0000  40.3399   0.6000
0.3296  -0.3153   9.1227   1.0000  -0.1805   5.6864   1.0000   0.0000
5   5  96.1871  93.7006  68.6860   0.0955  -0.4781   1.0000  17.8574   0.6000
0.2723  -0.2373   9.7875   1.0000  -0.0950   6.4757   1.0000   0.0000
6   6 109.1904  70.8314  30.0000   0.2765  -0.3000   1.0000  16.0000   0.1583
0.2804  -0.1994   8.1117   1.0000  -0.0675   8.2993   0.0000   0.0000
2   6 137.1002   0.0000   0.0000  -0.1902   0.0000   1.0000   6.0000   0.4256
17.7186   1.0000   0.0000   1.0000  -0.0377   6.4281   0.0000   0.0000
3   6 191.1743  52.0733  43.3991  -0.2584  -0.3000   1.0000  36.0000   0.8764
1.0248  -0.3658   4.2151   1.0000  -0.5004   4.2605   1.0000   0.0000
10     ! Nr of off-diagonal terms; Ediss;Ro;gamma;rsigma;rpi;rpi2
1   2   0.0591   1.7835   9.8002   1.0006  -1.0000  -1.0000   0.0000
2   3   0.0287   1.3294  10.5370   0.8004  -1.0000  -1.0000  523.4859
2   4   0.1099   1.8190   9.5142   1.2400  -1.0000  -1.0000  106.1598
1   3   0.1358   1.9395   9.1585   1.3715   1.1227   1.1482  113.7443
1   4   0.0286   1.4539  11.5574   1.2271   1.0011   1.1873  1615.1515
3   4   0.0250   1.7869  10.8055   1.4559   1.0878   1.0004   67.6876
2   6   0.0470   1.6738  11.6877   1.1931  -1.0000  -1.0000   0.0000
3   6   0.1263   1.8163  10.6833   1.6266   1.2052  -1.0000   0.0000
1   5   0.1408   1.8161   9.9393   1.7986   1.3021   1.4031   0.0000
2   5   0.0895   1.6239  10.0104   1.4640  -1.0000  -1.0000   0.0000
62     ! Nr of angles;at1;at2;at3;Thetao,o;ka;kb;pv1;pv2
1   1   1   70.6451   28.0854   1.1588   0.0000   0.1780   10.5736   1.0400
1   1   2   80.6132   17.9812   6.3171   0.0000   0.0591   0.0000   1.0400
```

2	1	2	79.9780	14.3158	1.3390	0.0000	0.0127	0.0000	1.0400
1	2	2	0.0000	0.0000	6.0000	0.0000	0.0000	0.0000	1.0400
1	2	1	0.0000	3.4110	7.7350	0.0000	0.0000	0.0000	1.0400
2	2	2	0.0000	27.9213	5.8635	0.0000	0.0000	0.0000	1.0400
1	1	3	60.6012	15.4885	6.7038	0.0000	0.1446	7.4341	1.1815
3	1	3	73.5573	26.1972	5.6525	0.0000	0.5602	-39.2416	2.0023
1	1	4	68.8624	35.0959	2.4661	0.0000	0.1057	19.4545	1.7357
3	1	4	67.6062	19.9590	3.3436	0.0000	3.6692	26.8252	1.4999
4	1	4	54.3806	23.0052	1.2131	0.0000	2.5573	0.0000	2.9925
2	1	3	74.8747	22.6563	2.9207	0.0000	0.9438	0.0000	1.6663
2	1	4	71.0777	9.1462	3.4142	0.0000	0.9110	0.0000	1.0400
1	2	4	0.0000	0.0019	6.3000	0.0000	0.0000	0.0000	1.0400
1	3	1	72.1018	38.4720	1.3926	0.0000	0.4785	0.0000	1.2984
1	3	3	89.9987	44.9806	0.5818	0.0000	0.7472	0.0000	1.2639
1	3	4	70.3281	12.9371	7.5000	0.0000	0.7472	0.0000	1.2639
3	3	3	84.2807	24.1938	2.1695	-10.0000	0.7472	0.0000	1.2639
3	3	4	84.2585	44.1039	0.9185	0.0000	0.7472	0.0000	1.2639
4	3	4	74.2312	25.7005	4.3943	0.0000	0.7472	0.0000	1.2639
1	3	2	84.1016	21.2916	2.5003	0.0000	0.7360	0.0000	2.9904
2	3	3	80.5517	10.0951	6.1566	0.0000	3.0000	0.0000	1.5219
2	3	4	73.9090	33.8700	6.3934	0.0000	2.1164	0.0000	1.4760
2	3	2	82.7429	36.2404	3.2589	0.0000	1.2996	0.0000	1.5608
1	4	1	72.5490	13.0651	1.6682	0.0000	2.6260	0.0000	1.5989
1	4	3	67.7338	30.0233	2.0265	0.0000	2.4507	0.0000	1.5485
1	4	4	60.3533	22.4389	9.6866	0.0000	2.1931	0.0000	0.8440
3	4	3	78.6753	49.8734	1.5058	-26.2246	1.8232	40.0000	3.2371
3	4	4	77.0399	35.8260	1.0222	-0.9193	2.4023	0.0000	1.6807
4	4	4	70.0790	21.6708	7.2553	0.0000	1.3863	0.0000	1.0440
1	4	2	71.7788	49.1169	0.8131	0.0000	0.3028	0.0000	1.4527
2	4	3	85.5146	48.1732	2.7880	0.0000	0.7990	0.0000	2.6735
2	4	4	72.2005	14.0699	2.9200	0.0000	0.4082	0.0000	2.6683
2	4	2	57.7399	17.0679	3.6793	0.0000	0.2900	0.0000	3.0237
1	2	3	0.0000	0.0019	6.0000	0.0000	0.0000	0.0000	1.0400
1	2	4	0.0000	0.0019	6.0000	0.0000	0.0000	0.0000	1.0400

1 2 5 0.0000 0.0019 6.0000 0.0000 0.0000 0.0000 1.0400
3 2 3 0.0000 0.1485 6.0000 0.0000 0.0000 0.0000 1.2958
3 2 4 0.0000 0.0074 6.0000 0.0000 0.0000 0.0000 1.4980 4 2 4 0.0000
0.0019 6.0000 0.0000 0.0000 0.0000 1.0400
2 2 3 0.0000 0.0019 6.0000 0.0000 0.0000 0.0000 1.0400
2 2 4 0.0000 0.0019 6.0000 0.0000 0.0000 0.0000 1.0400
1 1 5 74.4180 33.4273 1.7018 0.1463 0.5000 0.0000 1.6178
1 5 1 79.7037 28.2036 1.7073 0.1463 0.5000 0.0000 1.6453
2 1 5 63.3289 29.4225 2.1326 0.0000 0.5000 0.0000 3.0000
1 5 2 85.9449 38.3109 1.2492 0.0000 0.5000 0.0000 1.1000
1 5 5 85.6645 40.0000 2.9274 0.1463 0.5000 0.0000 1.3830
2 5 2 83.8555 5.1317 0.4377 0.0000 0.5000 0.0000 3.0000
2 5 5 97.0064 32.1121 2.0242 0.0000 0.5000 0.0000 2.8568
6 6 6 69.3456 21.7361 1.4283 0.0000 -0.2101 0.0000 1.3241
2 6 6 75.6168 21.5317 1.0435 0.0000 2.5179 0.0000 1.0400
2 6 2 78.3939 20.9772 0.8630 0.0000 2.8421 0.0000 1.0400
3 6 6 70.3016 15.4081 1.3267 0.0000 2.1459 0.0000 1.0400
2 6 3 73.8232 16.6592 3.7425 0.0000 0.8613 0.0000 1.0400
3 6 3 90.0344 7.7656 1.7264 0.0000 0.7689 0.0000 1.0400
6 3 6 22.1715 3.6615 0.3160 0.0000 4.1125 0.0000 1.0400
2 3 6 83.7634 5.6693 2.7780 0.0000 1.6982 0.0000 1.0400
3 3 6 73.4663 25.0761 0.9143 0.0000 2.2466 0.0000 1.0400
2 2 6 0.0000 47.1300 6.0000 0.0000 1.6371 0.0000 1.0400
6 2 6 0.0000 31.5209 6.0000 0.0000 1.6371 0.0000 1.0400
3 2 6 0.0000 31.0427 4.5625 0.0000 1.6371 0.0000 1.0400
2 2 5 0.0000 0.0019 6.0000 0.0000 0.0000 0.0000 1.0400
31 ! Nr of torsions;at1;at2;at3;at4;;V1;V2;V3;V2(BO);vconj;n.u;n
1 1 1 1 0.0000 48.4194 0.3163 -8.6506 -1.7255 0.0000 0.0000
1 1 1 2 0.0000 63.3484 0.2210 -8.8401 -1.8081 0.0000 0.0000
2 1 1 2 0.0000 45.2741 0.4171 -6.9800 -1.2359 0.0000 0.0000
0 1 2 0 0.0000 0.0000 0.0000 0.0000 0.0000 0.0000 0.0000
0 2 2 0 0.0000 0.0000 0.0000 0.0000 0.0000 0.0000 0.0000
0 1 3 0 1.7763 95.7472 0.3371 -4.2259 -2.0000 0.0000 0.0000
0 2 3 0 0.0000 0.1000 0.0200 -2.5415 0.0000 0.0000 0.0000 0 3 3 0

```
1.3153 116.0871    0.5104   -4.8204    0.0000    0.0000    0.0000
0  1  4  0  -1.3080 167.5582    0.1512   -4.1783   -0.0021    0.0000    0.0000
0  2  4  0    0.0000    0.1000    0.0200   -2.5415    0.0000    0.0000    0.0000
0  3  4  0    4.1603   27.6757    0.0186   -4.8563   -1.7500    0.0000    0.0000
0  4  4  0    2.9251   55.0854   -3.0688   -5.3950   -2.0000    0.0000    0.0000
0  1  1  0  -0.0247   25.7321   -1.4098   -9.0000   -1.0000    0.0000    0.0000
4  1  4  4  -1.9862   20.9784   -1.4653   -9.0000   -2.0000    0.0000    0.0000
0  1  5  0    4.0885   78.7058    0.1174   -2.1639    0.0000    0.0000    0.0000
0  5  5  0  -0.0170 -56.0786    0.6132   -2.2092    0.0000    0.0000    0.0000
0  2  5  0    0.0000    0.0000    0.0000    0.0000    0.0000    0.0000    0.0000
0  6  6  0    0.0000    0.0000    0.1200   -2.4426    0.0000    0.0000    0.0000
0  2  6  0    0.0000    0.0000    0.1200   -2.4847    0.0000    0.0000    0.0000
0  3  6  0    0.0000    0.0000    0.1200   -2.4703    0.0000    0.0000    0.0000
1  1  3  3    1.2707   21.6200    1.5000   -9.0000   -2.0000    0.0000    0.0000
1  3  3  1  -1.8804   79.9255   -1.5000   -4.1940   -2.0000    0.0000    0.0000
3  1  3  3  -2.0000   22.5092    1.5000   -8.9500   -2.0000    0.0000    0.0000
1  4  4  3    2.9552 131.2078    0.0042   -3.9717   -2.0000    0.0000    0.0000
1  1  3  4    1.2181 119.6186   -1.5000   -7.0635   -2.0000    0.0000    0.0000
2  1  3  4  -2.0000 156.6604    1.1004   -7.3729   -2.0000    0.0000    0.0000
1  3  4  3    2.3823 136.5288   -4.3308   -6.1290   -2.0000    0.0000    0.0000
1  1  4  2  -2.0000 147.2445   -1.5000   -7.0142   -2.0000    0.0000    0.0000
1  1  4  3  -2.0000   57.9085   -0.1690   -9.0000   -2.0000    0.0000    0.0000
2  3  4  3    0.7724 117.1943   -0.1249   -3.6148   -2.0000    0.0000    0.0000
2  4  4  3    0.0323   66.1401    0.4230   -3.6915   -2.0000    0.0000    0.0000
9     ! Nr of hydrogen bonds;at1;at2;at3;Rhb;Dehb;vhb1
3  2  3    2.0007   -8.1516    6.8955   20.2221
3  2  4    2.0432   -7.7406    3.6557   11.5797
4  2  3    1.8425   -0.3249    3.9503   10.9205
4  2  4    2.0005   -7.6051    4.1492   26.1996
3  2  5    2.6644   -3.0000    3.0000    3.0000
4  2  5    4.0476   -3.0000    3.0000    3.0000
5  2  3    2.1126   -4.5790    3.0000    3.0000
5  2  4    2.2066   -5.7038    3.0000    3.0000
5  2  5    1.9461   -4.0000    3.0000    3.0000
```

Training set

The training set includes the following data:

(1) The geometries (bond length, valence angles and dihedral angles) of common small molecules (such as water, nitrogen, hydrogen, oxygen, ammonia, carbon monoxide, carbon dioxide and etc.) and energetic materials (HMX, TATB, nitromethane and etc.).

(2) The bond dissociation and compression curves, valence angles bending curves, dihedral angle torsion curves of common small molecules.

(3) The geometries of reactant, intermediates and products, including all minimum points from decomposition paths of NTO, RDX and FOX-7 [13,26-27]. A full decomposition path were provided in the file "decomposition paths of NTO.jpg".

(4) The compressed and expanded equation of states for NTO, RDX and FOX-7 ($0.85V_0 - 1.2V_0$,V_0 was the initial unit cell volume).

(5) The bond dissociation and compression curves, valence angles bending curves, dihedral angle torsion curves of energetic materials (NTO, RDX, FOX-7, nitromethane and etc.).

(6) The reaction potentials energy surfaces of unimolecular decomposition paths [13,26-27] of NTO, RDX and FOX-7.

(7) The covalent bond energies under different chemical environments.

(8) The NO_2 dissociation energies of HMX, TATB, DNT and etc.

(9) The interaction energy of dimers and trimers for NTO and FOX-7.

The completed training-data files can be requested by contacting the author. Two email address are provided here: 3120185542@bit.edu.cn dulixiaosong@163.com

Table S1. Shock Hugoniot data of α-NTO

U_P (km/s)	U_s (km/s)	V/V_0	Calculation Pressures (GPa)
2.0	0.24	0.878	0.919
3.0	0.60	0.799	3.447
3.5	0.89	0.747	5.966
4.0	1.09	0.727	8.351
4.5	1.33	0.704	11.464
5.0	1.59	0.681	15.23
5.5	1.88	0.659	19.801
6.0	2.22	0.630	25.510
7.0	2.90	0.585	38.882
8.0	3.56	0.555	54.552
9.0	4.27	0.525	73.614
10.0	5.17	0.483	99.026
11.0	5.89	0.464	124.154

Animations and discussion of the phenomenon mentioned in chapter 3.1.2

The noticeable phenomenon observed from the original ReaxFF-lg MD was that the hydrogen atoms in the nitrogen heterocycle ring were not on the same plane as the ring, but this phenomenon did not happen in the new ReaxFF-lg MD. For detail information about this phenomenon, we provided a few animations in here: the file named as "newReaxFF.avi" is the dynamic results by new ReaxFF-lg potential; the file named as "ReaxFF_liu.avi" is the dynamic results by original ReaxFF-lg potential.

We also count the change curve of the corresponding bond distance and dihedral angle in the MD process, as shown in the Figure below:

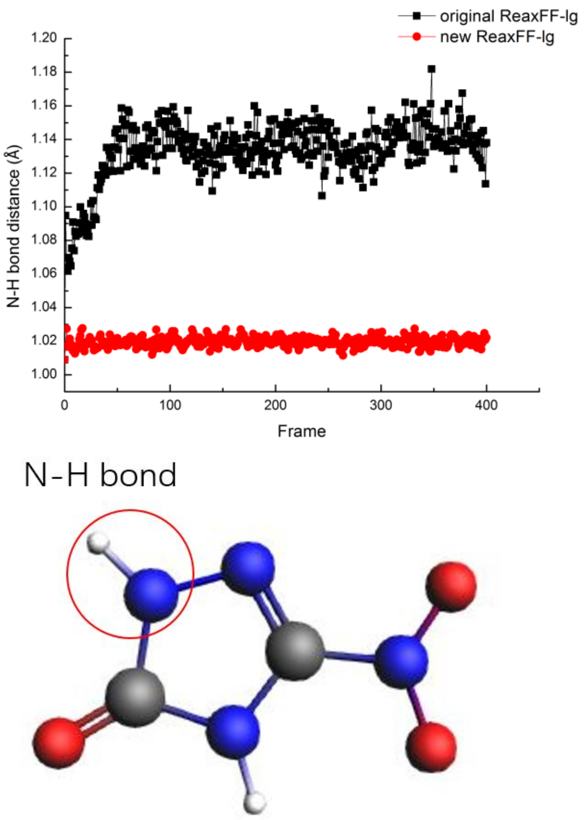

Figure S1. N-H bond distance during NVT-MD.

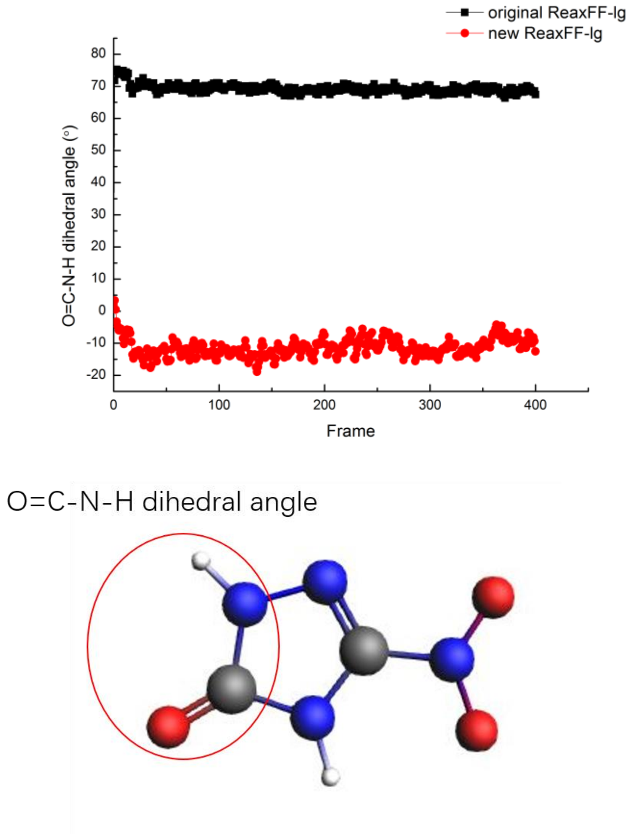

Figure S2. O=C-N-H bond distance during NVT-MD.

From Figure 1-2, we found that the N-H bond distance and O=C-N-H dihedral angle obtained from new ReaxFF-lg MD behave less change than original ReaxFF-lg MD, and is Also more consisency with the original structure. But the original ReaxFF-lg potentials totally failed to reproduce the initial O=C-N-H dihedral during MD, and the predicted N-H bond distance is 0.1 angstroms larger than the original structure. So we think the original ReaxFF-lg should not be used for simulating the NTO crystals.
