# Supplementary figures and images for: Initial Decomposition Mechanism of 3-Nitro-1,2,4-triazol-5-one (NTO) under Shock Loading: ReaxFF Parameterization and Molecular Dynamic Study
Source: Molecules. 2021 Aug 9;26(16):4808. doi: 10.3390/molecules26164808 (PMC8400475; doi:10.3390/molecules26164808)

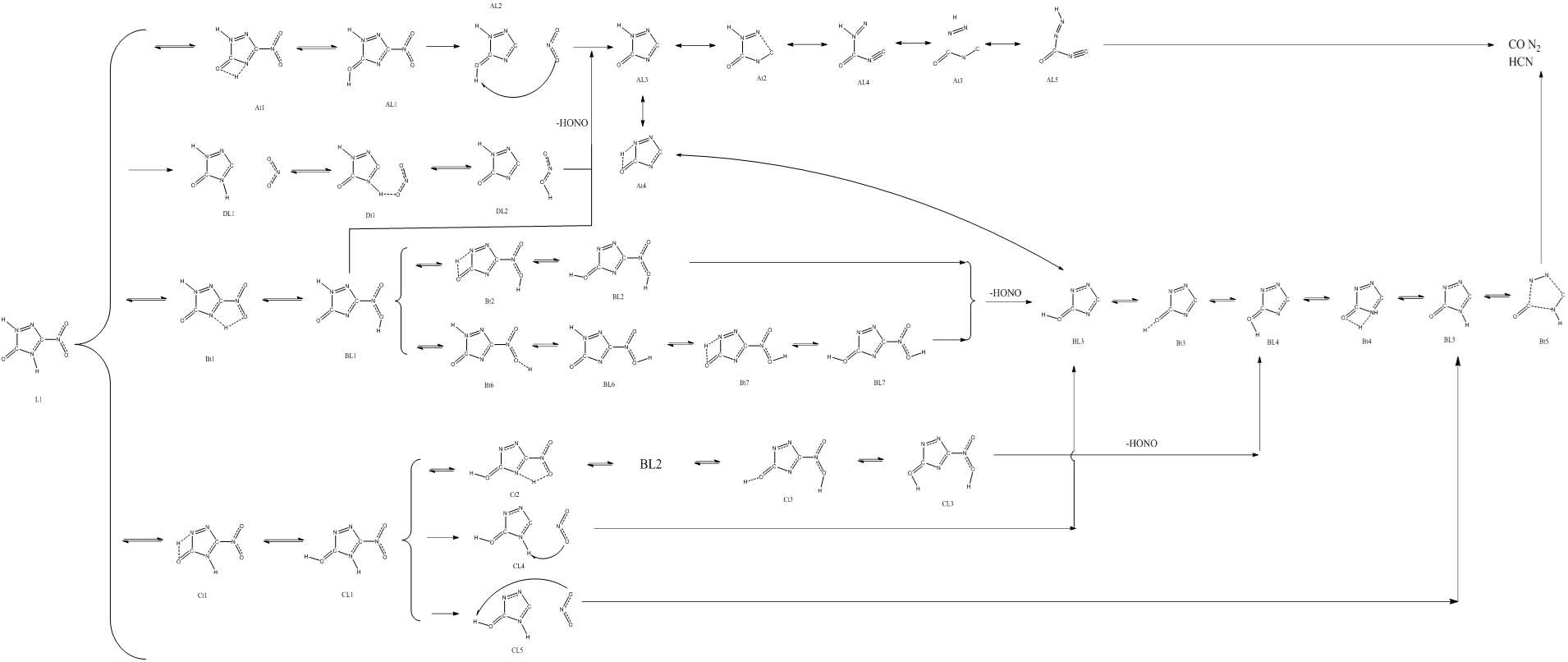

Supplement: Supplementary file 1 [file molecules-26-04808-s001.zip › decomposition paths of NTO.tif]
